# New Mechanistic Insights into Purine Biosynthesis with Second Messenger c-di-AMP in Relation to Biofilm-Related Persistent Methicillin-Resistant Staphylococcus aureus Infections

**DOI:** 10.1128/mBio.02081-21

**Published:** 2021-11-02

**Authors:** Liang Li, Yi Li, Fengli Zhu, Ambrose L. Cheung, Genzhu Wang, Guangchun Bai, Richard A. Proctor, Michael R. Yeaman, Arnold S. Bayer, Yan Q. Xiong

**Affiliations:** a The Lundquist Institute for Biomedical Innovation, Harbor-UCLA Medical Center, Torrance, California, USA; b Geisel School of Medicine at Dartmouth, Hanover, New Hampshire, USA; c Department of Immunology and Microbial Diseases, MC-151, Albany Medical College, Albany, New York, USA; d Departments of Medicine and Medical Microbiology/Immunology, University of Wisconsin School of Medicine and Public Health, Madison, Wisconsin, USA; e David Geffen School of Medicine at UCLA, Los Angeles, California, USA; University of Rochester

**Keywords:** MRSA, c-di-AMP, purine biosynthesis, biofilm, vancomycin, persistence, endovascular infection

## Abstract

Persistent methicillin-resistant Staphylococcus aureus (MRSA) endovascular infections represent a significant clinically challenging subset of invasive, life-threatening S. aureus infections. We have recently demonstrated that purine biosynthesis plays an important role in such persistent infections. Cyclic di-AMP (c-di-AMP) is an essential and ubiquitous second messenger that regulates many cellular pathways in bacteria. However, whether there is a regulatory connection between the purine biosynthesis pathway and c-di-AMP impacting persistent outcomes was not known. Here, we demonstrated that the purine biosynthesis mutant MRSA strain, the Δ*purF* strain (compared to its isogenic parental strain), exhibited the following significant differences *in vitro*: (i) lower ADP, ATP, and c-di-AMP levels; (ii) less biofilm formation with decreased extracellular DNA (eDNA) levels and Triton X-100-induced autolysis paralleling enhanced expressions of the biofilm formation-related two-component regulatory system *lytSR* and its downstream gene *lrgB*; (iii) increased vancomycin (VAN)-binding and VAN-induced lysis; and (iv) decreased wall teichoic acid (WTA) levels and expression of the WTA biosynthesis-related gene, *tarH*. Substantiating these data, the *dacA* (encoding diadenylate cyclase enzyme required for c-di-AMP synthesis) mutant strain (*dacA_G206S_* strain versus its isogenic wild-type MRSA and *dacA*-complemented strains) showed significantly decreased c-di-AMP levels, similar *in vitro* effects as seen above for the *purF* mutant and hypersusceptible to VAN treatment in an experimental biofilm-related MRSA endovascular infection model. These results reveal an important intersection between purine biosynthesis and c-di-AMP that contributes to biofilm-associated persistence in MRSA endovascular infections. This signaling pathway represents a logical therapeutic target against persistent MRSA infections.

## INTRODUCTION

Persistent methicillin-resistant Staphylococcus aureus (MRSA) bacteremia (PB) (defined as ≥7 days of positive blood cultures despite appropriate antibiotic therapy) represents up to 30% of S. aureus endovascular infections (1, 2). Importantly, many PB clinical isolates are susceptible *in vitro* to anti-MRSA agents, such as vancomycin (VAN) and daptomycin (DAP), by Clinical and Laboratory Standards Institute (CLSI) standards, yet they persist *in vivo* (2, 3). Therefore, persistent MRSA infections pose an unmet therapeutic challenge, and understanding the specific molecular mechanisms involved in these outcomes are essential for their ultimate successful treatment.

S. aureus strains employ a wide variety of small nucleotide-signaling molecules that allow them to adjust their cellular physiology to cope with unfavorable environmental conditions for survival (4, 5). Among these molecules, the recently discovered cyclic di-AMP (c-di-AMP) is an essential and ubiquitous second messenger in Gram-positive bacteria (6–8). In S. aureus, c-di-AMP is synthesized from two molecules of ATP via a complex pathway involving the diadenylate cyclase enzyme, DacA (encoded by *dacA*) and the phosphodiesterase enzyme, GdpP (encoded by *gdpP*) (6, 9). Although the role of c-di-AMP in bacterial cell physiology, biofilm formation, adaptation to environmental stresses, and virulence has been reported (6, 7, 9–12), little is known about its impact on the persistent outcomes in MRSA endovascular infections.

Recently, we discovered an important role of purine biosynthesis in staphylococcal cell growth, regulation of global regulators, and the stringent response, which contributes significantly to persistent outcomes in MRSA endovascular infections (13, 14). It is well known that purine biosynthesis is crucial for cell growth through DNA and RNA synthesis, as well as ATP energy supply (15). Interestingly, as a second messenger, c-di-AMP, synthesized from nucleotide ATP, is also critical for bacterial cell growth and has interconnections with the stringent response (6, 9). Hence, we hypothesized that purine biosynthesis and c-di-AMP regulation may positively bias toward persistent phenotypes (e.g., biofilm formation and persistence to cell wall-active anti-MRSA antibiotics [16, 17]). The current investigation was designed to explore this hypothesis relating purine biosynthesis, c-di-AMP, and the persistent outcomes in MRSA endovascular infections both *in vitro* and in an experimental endocarditis model.

## RESULTS

### *purF* and c-di-AMP participate in a coordinated network.

**FIG 1:**
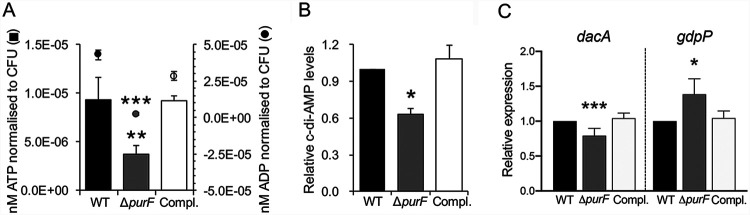
The *purF* mutant had significantly lower intracellular ATP and ADP levels (A), impaired c-di-AMP levels (B), and a relative lower expression of *dacA* and higher expression of *gdpP* (C) versus its isogenic JE2 MRSA strain (WT) and *purF*-complemented strains (Compl.). Relative expression levels of *dacA* and *gdpP* were calculated by normalizing the expression level of each gene versus housekeeping gene *gyrB*. All experiments were performed independently at least twice with triplicates (*n* ≥ 6). Error bars represent standard deviations. *, *P* < 0.05; **, *P* < 0.01; ***, *P* < 0.001 (*purF* mutant versus WT and *purF*-complemented strains).

We recently demonstrated that clinical PB strains (versus resolving MRSA bacteremia [RB] strains, defined as isolates from patients with negative blood cultures 2 to 4 days after initiation of therapy, [1, 2]) showed significantly higher expression of purine biosynthesis pathway genes, including *purF* (18). In addition, purine biosynthesis produces critical substrates for c-di-AMP synthesis (i.e., ATP), thus raising the possibility of a correlation between these events (6). To assess the role of purine biosynthesis in cellular c-di-AMP production, MRSA parental strain JE2 and its isogenic *purF* mutant and *purF-*complemented strains were employed in this study. Decreased levels of ATP, ADP, and c-di-AMP were found in the *purF* mutant strain when compared with those of its parental and *purF*-complemented strains (Fig. 1A and B, respectively). In S. aureus, c-di-AMP is synthesized by DacA and hydrolyzed by GdpP (9). To examine whether the purine biosynthesis pathway coordinates the transcription of c-di-AMP-related genes, *dacA* and *gdpP* expressions were also determined. Significantly decreased *dacA* and increased *gdpP* expressions were found in the *purF* mutant versus those in its isogenic JE2 parental and *purF*-complemented strains (Fig. 1C). These results support a unique and coordinated network connecting purine biosynthesis and c-di-AMP production.

### c-di-AMP promotes *purF*-mediated biofilm formation, extracellular DNA levels, and autolysis activity.

**FIG 2:**
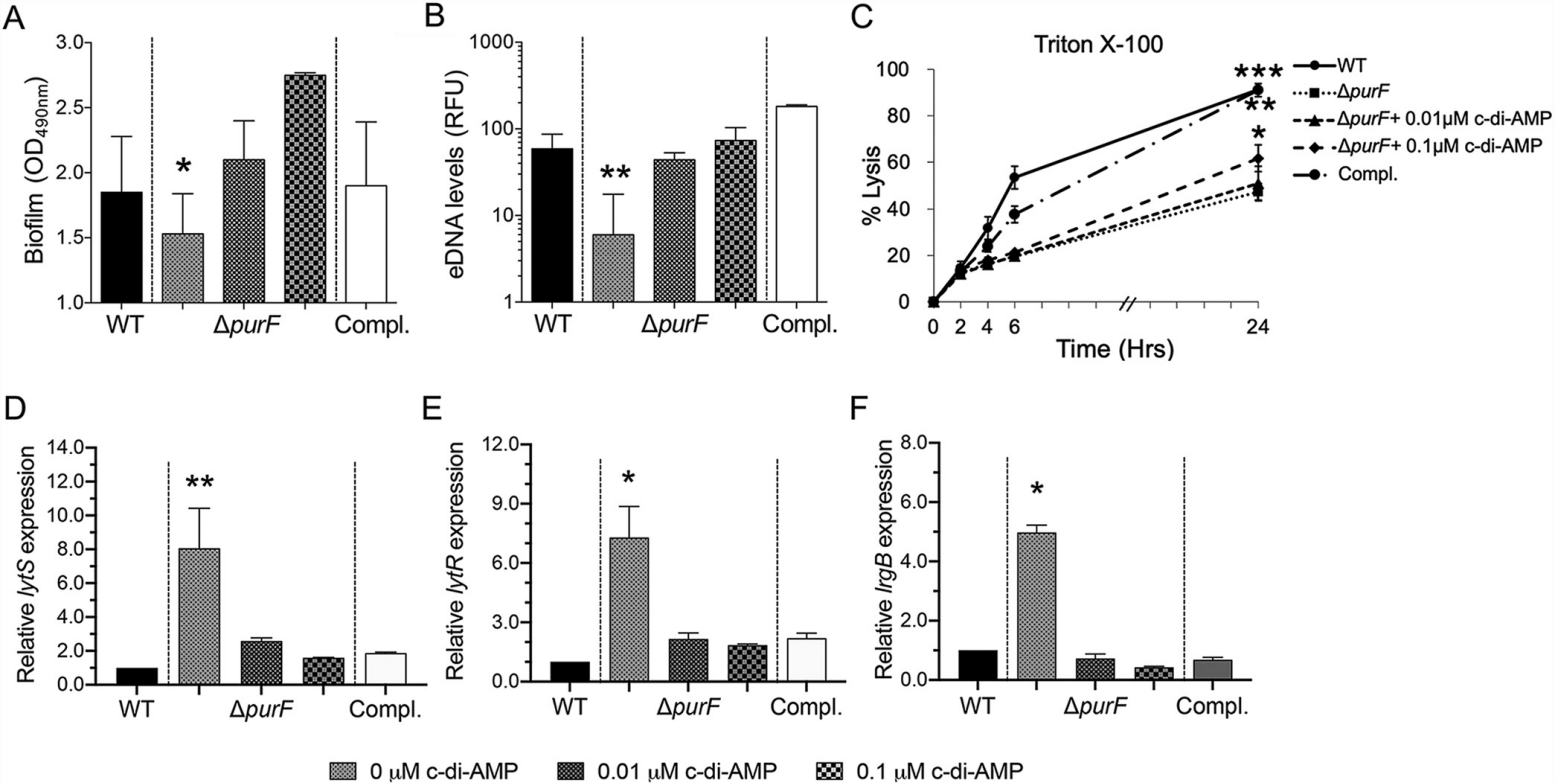
The *purF* mutant showed decreased biofilm formation (A), eDNA levels (B), and Triton X-100-induced autolysis (C) and increased expression of *lytS* (D), *lytR* (E), and *lrgB* (F) versus its MRSA JE2 parental (WT) and *purF*-complemented (Compl.) strains. The impact of *purF* on these profiles could be reversed by addition of c-di-AMP. The relative expression levels of *lytS*, *lytR*, and *lrgB* were calculated by normalizing the expression level of each gene versus housekeeping gene *gyrB*. All experiments were performed independently at least twice with triplicates (*n* ≥ 6). Error bars represent standard deviations. *, *P* < 0.05; **, *P* < 0.01; ***, *P* < 0.001 (*purF* mutant versus WT and *purF*-complemented strains and groups with additional c-di-AMP exposure).

The *purF* mutant strain exhibited significantly less biofilm formation versus its isogenic MRSA JE2 parental and *purF*-complemented strains (Fig. 2A). The impaired biofilm formation in the *purF* mutant could be fully restored by adding exogenous 0.01 μM c-di-AMP (Fig. 2A). In addition, it is known that released extracellular DNA (eDNA) in the biofilm matrix derived from genomic DNA promotes biofilm formation; a potential source for this eDNA derives from cell lysis (19–21). Supporting this concept, significantly decreased eDNA levels (∼7.0-fold) paralleling an ∼6.5-fold reduction of Triton X-100-induced cell lysis (at 24-h exposure) were observed within the *purF* mutant compared to those in its isogenic JE2 parental and *purF*-complemented strains (Fig. 2B and C, respectively).

As it has been reported, one of the two-component signal transduction systems, *lytSR*, plays an important role in biofilm development (22) and regulates the expression of *lrgB*, which negatively correlates with eDNA levels, cell lysis, and biofilm formation in S. aureus (23). Therefore, we evaluated *lytSR* and *lrgB* expression to establish whether the observed effects in eDNA and autolysis as above were attributable to altered *lytSR* and *lrgB* transcriptions in the JE2 strain set. Indeed, there was a reciprocal relationship between the loss of *purF* function and gain of *lytSR* and *lrgB* expressions. The *purF* mutant exhibited significantly increased *lytSR* and *lrgB* expressions versus those of its JE2 parental and *purF*-complemented strains (Fig. 2D to F). Notably, the addition of exogenous c-di-AMP significantly increased eDNA levels and cell lysis activity (Fig. 2B and C) and decreased *lytSR* and *lrgB* expressions in the *purF* mutant strain in a concentration-dependent manner (Fig. 2D to F). Taken together, these results demonstrated that purine biosynthesis positively regulates biofilm formation, eDNA levels, and autolysis activity but negatively impacts *lytSR* and *lrgB* expressions through a mechanism that is modulated by c-di-AMP.

### *purF* is involved in VAN-MRSA binding and ensuing lysis.

**FIG 3:**
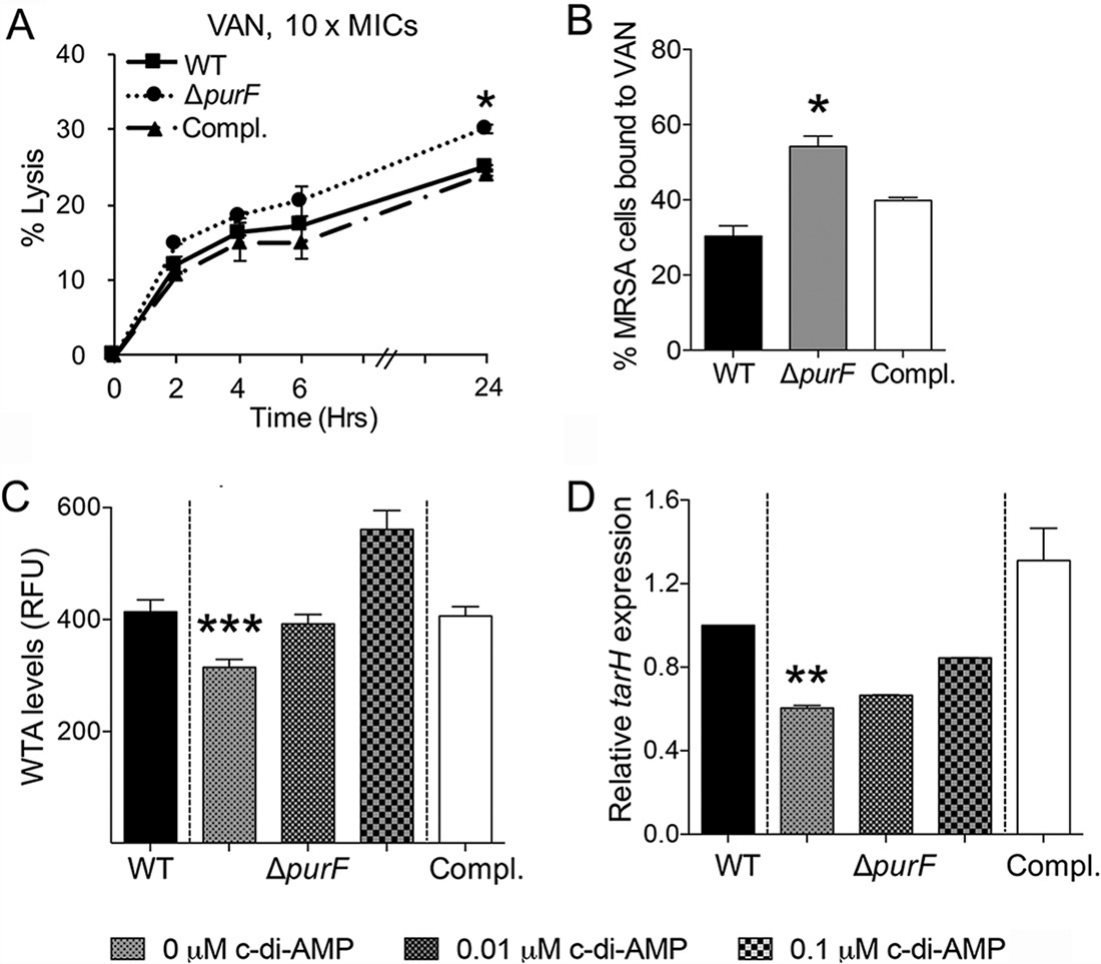
The *purF* mutant showed enhanced VAN-induced lysis (A) and VAN binding (B), lower WTA levels (C), and *tarH* expression (D) versus JE2 parental (WT) and *purF*-complemented (Compl.) strains. The impact of *purF* on these profiles could be reversed by addition of c-di-AMP. The relative expression level of *tarH* was calculated by normalizing the expression level of *tarH* versus housekeeping gene *gyrB*. All experiments were performed independently at least twice with triplicates (*n* ≥ 6). Error bars represent standard deviations. *, *P* < 0.05; **, *P* < 0.01; ***, *P* < 0.001 (*purF* mutant versus WT and *purF*-complemented strains and groups with additional c-di-AMP exposure).

Our previous work showed that the JE2 parental, its isogenic *purF* mutant, and *purF*-complemented strains have identical VAN MICs (2 μg/ml) (13). However, the *purF* mutant exhibited significantly lower *in vitro* survival rates with VAN exposure under *in vivo*-like conditions than its JE2 parental and *purF*-complemented strains (13). Thus, we investigated the impact of purine biosynthesis on VAN-induced lysis of this JE2 strain set. In the presence of 10× MIC of VAN (20 μg/ml), the *purF* mutant showed a significantly higher percentage of lysis than the JE2 parental or *purF*-complemented strains at the 24-h time point (Fig. 3A).

In S. aureus, VAN inhibits cell wall synthesis by binding to the terminal d-Ala-d-Ala of peptidoglycan (24). Therefore, we hypothesized that the enhanced susceptibility to VAN would correlate with VAN-MRSA binding. Indeed, a significantly higher percentage of VAN binding was observed in the *purF* mutant strain (54%) than in its isogenic JE2 parental (30%) and *purF*-complemented (40%) strains (Fig. 3B).

### *purF* and c-di-AMP promote WTA and *tarH*-mediated integrity.

Next, we explored the hypothesis that *purF* and c-di-AMP influence cell wall composition and integrity. In S. aureus, cell wall teichoic acid (WTA) serves as an integral component of the cell wall and has been reported to alter VAN susceptibility in S. aureus (25, 26). Thus, we quantified the relationship between *purF* and c-di-AMP on WTA levels to assess the impact of purine biosynthesis and c-di-AMP regulation on WTA levels. Significantly reduced WTA levels were observed in the *purF* mutant versus its JE2 parental and *purF*-complemented strains (Fig. 3C). These data suggested that repression of purine biosynthesis can lead to a reduction of WTA levels. To further define the mechanisms underlying the WTA-related phenotypes, we focused on the expression of *tarH* in the study strain set, which is positively correlated with WTA biosynthesis (27). In the *purF* mutant, transcription of *tarH* was markedly diminished compared with that of its isogenic parental JE2 and *purF*-complemented strains (Fig. 3D). Importantly, exposure to exogenous c-di-AMP increased WTA levels and *tarH* expression in the *purF* mutant strain in a concentration-dependent manner (Fig. 3C and D).

Collectively, these data indicated that purine biosynthesis contributes to VAN persistence via cell binding and induced lysis, corresponding to WTA levels and *tarH* expression. Because exogenous c-di-AMP reverses these effects, these data also points to a novel mechanistic relationship among purine biosynthesis, c-di-AMP, and WTA levels, which may contribute to persistent outcomes.

### The roles of c-di-AMP in the JE2 background are validated in a distinct MRSA background.

**FIG 4:**
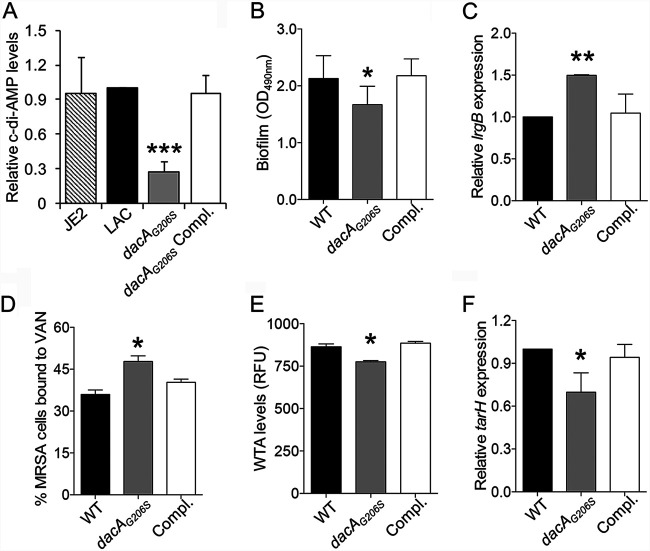
The *dacA_G206S_* mutant had a similar impact on biofilm formation, *lrgB* expression, VAN binding, WTA levels, and *tarH* expression as the *purF* mutant strain. Intracellular c-di-AMP levels (A), biofilm formation (B), relative expression of *lrgB* (C), VAN binding (D), WTA levels (E), and relative expression of *tarH* (F) in the MRSA LAC parental strain (WT), its isogenic *dacA_G206S_* mutant strain (*dacA_G206S_*), and the *dacA*-complemented (Compl.) strain. The relative expression levels of *lrgB* and *tarH* were calculated by normalizing the expression level of each gene versus housekeeping gene *gyrB*. All experiments were performed independently at least twice with triplicates (*n* ≥ 6). Error bars represent standard deviations. *, *P* < 0.05; **, *P* < 0.01; ***, *P* < 0.001 (*dacA_G206S_* mutant versus WT and *dacA-*complemented strain).

It is essential to demonstrate that the above findings are not restricted to one S. aureus genetic background; hence, MRSA parental strain LAC parental, its isogenic *dacA_G206S_* mutant, and *dacA*-complemented strains were employed. First, we demonstrated that there were no significant differences in c-di-AMP levels between the LAC parental strain and its plasmid-cured derivation strain JE2 parental, which has been used above (Fig. 4A). Consistent with previously published results (6), the *dacA_G206S_* mutant showed significantly lower c-di-AMP levels compared to those of its parental strain LAC and the *dacA*-complemented variant (Fig. 4A). Despite all of the MRSA isolates having a VAN-susceptible phenotype (MICs = 2 μg/ml), the *dacA_G206S_* mutant exhibited significantly (i) reduced biofilm formation paralleling increased *lrgB* expression (Fig. 4B and C, respectively), (ii) increased VAN binding (Fig. 4D), and (iii) decreased WTA levels with lower *tarH* expression (Fig. 4E and F, respectively). Of note, results observed in the *dacA_G206S_* mutant could be partially restored by the addition of exogenous c-di-AMP at the concentration of 0.01 μM or 0.1 μM (data not shown).

### c-di-AMP contributes to VAN persistence in a rabbit model of endocarditis.

**FIG 5:**
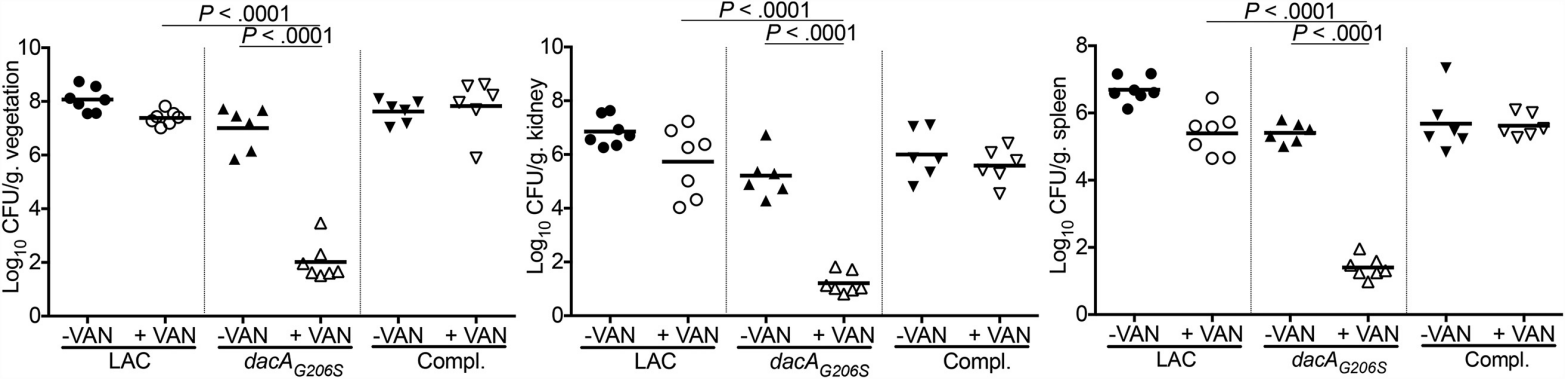
The *dacA_G206S_* mutant significantly enhanced the efficacy of VAN in a rabbit endocarditis model. Densities of MRSA in target tissues in the endocarditis model due to 10^5^ CFU challenges of the LAC parental strain, its isogenic *dacA_G206S_* mutant (*dacA_G206S_*), or *dacA*-complemented (Compl.) strain with/without VAN treatment. Each dot represents one animal. Horizontal black bars indicate means of MRSA densities.

Our findings above demonstrated a functional relationship among purine biosynthesis, c-di-AMP, VAN-MRSA binding and lysis, and biofilm formation. Together, these observations strongly supported our hypothesis that the interaction of purine biosynthesis and c-di-AMP contributes to persistent outcomes during VAN therapy *in vivo*. Therefore, to assess the putative impact of c-di-AMP on the persistent outcomes *in vivo*, an experimental infective endocarditis model was used. At baseline (without VAN therapy), the *dacA_G206S_* mutant had similar MRSA counts in vegetations but lower counts in kidney and spleen compared to its parental LAC or *dacA*-complemented strains (Fig. 5). Importantly, animals infected by the *dacA_G206S_* mutant were hypersusceptible to VAN treatment, with significantly reduced MRSA densities (<3.5 log_10_ CFU/g tissue) in all target tissues compared to those of the parental or *dacA*-complemented strains (Fig. 5). These *in vivo* data affirmed that reduced c-di-AMP levels are associated with enhanced VAN responsiveness in the endocarditis model.

## DISCUSSION

**FIG 6:**
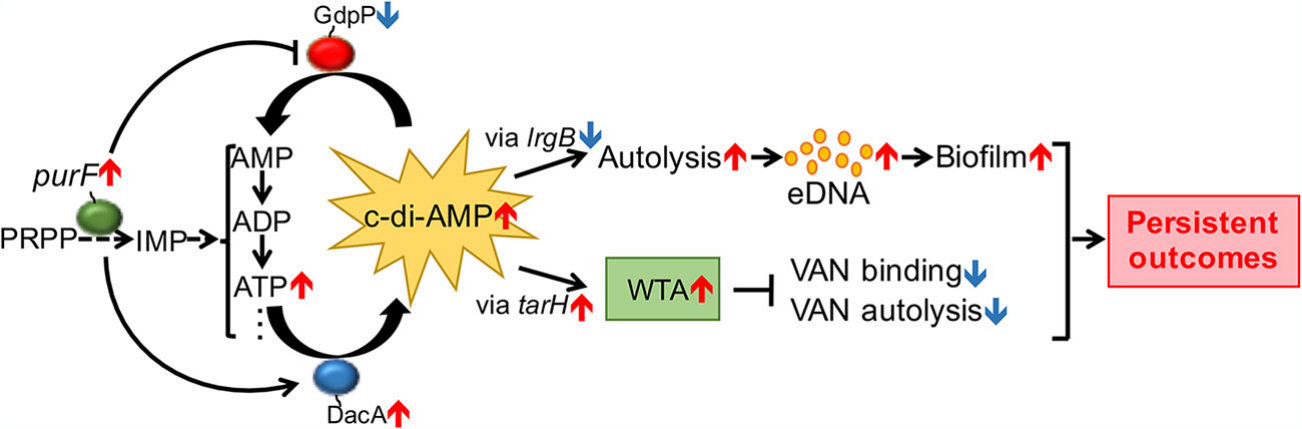
Model depicting the role of the second messenger c-di-AMP through purine biosynthesis in persistent MRSA endovascular infection. As a rate-limit enzyme, the ATase (PRPP→PRA)-encoding gene *purF* participates in turning 5-phosphoribosyl-1-pyrophosphate (PRPP) into IMP in the purine biosynthesis pathway (15). The branch-point intermediate IMP ultimately converts to essential nucleobases adenine including AMP, ADP, and ATP (55). c-di-AMP is synthesized from two molecules of ATP through the diadenylate cyclase enzyme DacA and hydrolyzed by the phosphodiesterase enzyme GdpP (6, 9). In the current study, purine biosynthesis was shown to contribute to the induction and repression of the expression of *dacA* and *gdpP*, respectively, and then subsequently elevated c-di-AMP levels. Increased c-di-AMP benefits persistent-related factors, such as (i) downregulation of *lrgB* expression, which leads to increased lysis, eDNA, and biofilm formation; and (ii) upregulation of *tarH* expression, which results in higher WTA levels, and subsequent decreased VAN-induced lysis and VAN-binding, thus ultimately facilitating persistent outcomes.

The regulatory intersection between purine biosynthesis and c-di-AMP generation in persistent MRSA endovascular infections is not well studied. The present study assessed this interrelationship and its key downstream impacts on phenotypes relevant to persistent outcome in MRSA infections. Our study demonstrated that purine biosynthesis positively regulates c-di-AMP synthesis, which can ultimately modulate biofilm formation, WTA levels, VAN binding, and lysis and contribute to the persistent MRSA endovascular infections (Fig. 6).

A positive regulatory correlation between purine biosynthesis and c-di-AMP was demonstrated in our study strain set. For instance, significantly decreased *dacA* (encoding c-di-AMP synthesis enzyme) and increased *gdpP* (encoding c-di-AMP hydrolysis enzyme) expression levels were found in the *purF* mutant versus its parental and *purF*-complemented strains. Consistent with our current findings, DeFrancesco et al. also reported a positive relationship between purine biosynthesis and c-di-AMP, showing that deletion of the purine biosynthesis repressor, PurR, was associated with increased c-di-AMP levels in S. aureus (19). Therefore, it appears that any conditions significantly affecting purine biosynthesis may consequently impact c-di-AMP levels.

Biofilm formation, a major virulence factor in medical device-related S. aureus endovascular infections, accelerates bacterial colonization in host tissues and promotes resistance to host immune responses and antimicrobial agents (16, 28). The role of c-di-AMP on biofilm formation has been studied in S. aureus (29) and Streptococcus mutans (10). In the current study, our data revealed that the biofilm formation significantly decreased in the *purF* mutant was restored by the addition of exogenous c-di-AMP. This finding indicates a dependent effect of purine biosynthesis on c-di-AMP in the biofilm formation. Similar to c-di-AMP, the second messenger c-di-GMP has also been shown to affect biofilm formation in bacteria (30). However, no quantifiable amounts of c-di-GMP could be detected in the JE2 strain set after multiple quantification attempts (data not shown). These results are consistent as previously reported by Corrigan et al. (29) and Holland et al. (31) showing that c-di-GMP levels are not detectable in the study S. aureus strains. Thus, c-di-GMP appears unlikely to be involved in biofilm formation in the S. aureus strains tested. Release of eDNA occurs through autolysis during programmed cell death (23, 32, 33). Dengler et al. reported that a *dacA* mutation decreased c-di-AMP levels, resulting in reduced autolysis (Triton X-100) in S. aureus (7). In line with this report, we demonstrated that lower c-di-AMP levels in the *purF* mutant correlated with decreased eDNA versus that of its parental and *purF*-complemented strains. In contrast, DeFrancesco et al. observed an opposite result, that disruption of GdpP yielded increased c-di-AMP but lower eDNA levels (19). Our results revealed that the reduced eDNA levels in the *purF* mutant were partially restored by exogenous c-di-AMP exposure at a concentration of 0.01 μM or 0.1 μM. Interestingly, however, a higher c-di-AMP concentration (1 μM) exposure led to an opposite effect in the *purF* mutant, corresponding with reduced eDNA (data not shown); thus, these contrasting findings may be due to an “overdose” of intracellular c-di-AMP. In parallel, the two-component regulatory system *lytSR* and its downstream *lrgB* operon are known to be involved in biofilm formation by controlling cell lysis and releasing eDNA in S. aureus (22, 34). To date, it has been established that the *lytSR* two-component regulatory system positively regulates *lrgAB* transcriptions (22, 35). In addition, Beltrame et al. reported a direct correlation between the expression of *lrgB* and eDNA levels (23). Interestingly, the current study demonstrated that the *purF* mutant had a significantly lower c-di-AMP level but increased *lytSR* and *lrgB* expressions. In turn, this effect led to the decreased cell lysis and eDNA release, corresponding to reduced biofilm formation (versus the parental and *purF*-complemented strains). Importantly, the isogenic strain defective in c-di-AMP synthesis (i.e., *dacA_G206S_* mutant) confirmed the correlation among c-di-AMP, *lrgB* expression, and biofilm formation. These results provide a plausible linkage among purine biosynthesis, c-di-AMP generation, *lytSR* and *lrgB* expressions, and biofilm formation. This novel relationship has not been previously identified and is likely to contribute to *in vivo* persistent outcomes in S. aureus infection.

VAN was selected in our study since it has remained the gold standard for the treatment of invasive MRSA infections. Peschel et al. reported that the level of WTA is inversely associated with VAN binding, cell lysis, and subsequently VAN susceptibility in S. aureus (26). We studied the potential impact of purine biosynthesis and c-di-AMP in this regard. Prior results support our current findings, demonstrating significantly lower WTA levels and higher VAN binding and VAN-induced lysis in the *purF* mutant versus its parental strain. The addition of c-di-AMP restored WTA levels that were decreased in the *purF* mutant strain, providing mechanistic confirmation of this phenotype. These results further support an important linkage among purine biosynthesis, c-di-AMP, and WTA synthesis, which influences VAN susceptibility related to persistence. Next, we explored the impact of *tarH* expression on WTA composition. TarH is part of the two-component ATP-binding cassette (ABC) transporter, TarGH, which is responsible for the translocation of WTA through the cell membrane (36). Thus, *tarH* expression directly affects WTA levels in S. aureus (37). A positive relationship between WTA levels and *tarH* transcription has been previously reported (27), consistent with our results showing a decreased *tarH* expression and WTA levels in the *purF* mutant versus those in its isogenic parental strain. Importantly, exogenous c-di-AMP reversed this effect, restoring *tarH* expression in the *purF* mutant strain. Unlike the expression of *tarH*, no significant differences in *tarG* expression were observed between the *purF* and *dacA_G206S_* mutant strains versus their respective parental and complementary strains (data not shown). These results are consistent with the previous observation by Wanner et al. (27) showing that, among the analyzed WTA biosynthesis genes (*tarO*, *tarA*, *tarK*, *tarL*, *tarG*, and *tarH*), only *tarH* transcription was significantly increased in WTA-elevated S. aureus. Therefore, these results suggest that the impact of c-di-AMP on WTA synthesis might be mainly through regulating *tarH* expression. Furthermore, the impacts of c-di-AMP on these *in vitro* phenotypic and genotypic profiles related to persistence (e.g., biofilm formation, VAN binding, and WTA synthesis) were confirmed in a genetically defined strain set, including the MRSA LAC parental strain and its isogenic *dacA_G206S_* mutant strain. These findings are supported by a previous study demonstrating that c-di-AMP is involved in cell envelope signaling and can influence cell wall-active antibiotic resistance in S. aureus (7). Collectively, these results uncovered the interactions among purine biosynthesis pathway, c-di-AMP, and persistence related profiles.

The impacts of c-di-AMP in bacterial pathogenesis have been noted in experimental murine lung and skin infection models due to Mycobacterium tuberculosis and Streptococcus pyogenes, respectively (38, 39). In our current investigations, animals infected with the *dacA_G206S_* mutant were significantly more susceptible to VAN treatment than those infected with the parental strain in experimental endocarditis. These outcomes suggest that the *in vivo* effect of c-di-AMP in VAN persistence in MRSA may be due, at least in part, to a combination of impacts on biofilm formation, WTA levels, VAN binding, and VAN-induced lysis.

We recognize that there were certain limitations in the current study. First, we only studied one MRSA genetic background strain set. However, validation of the primary findings in the JE2 strain set using a predominant clinical isolate MRSA strain set (LAC) supports the key concepts of the current study. Nonetheless, it is possible that the regulation of this important network may differ somewhat in other MRSA genetic background. Second, we understand that many other factors may also impact c-di-AMP generation and purine biosynthesis, which could contribute to persistent MRSA infections (e.g., other cell wall components, such as peptidoglycan [29], or host anti-inflammatory response, such as macrophage, etc. [11]). Such factors are certainly priorities for investigations beyond the scope of the current effort. Lastly, determining the detailed mechanisms of how c-di-AMP regulates autolysis and WTA are ongoing in our laboratories.

In summary, the present findings are the first to our knowledge to demonstrate the interaction between purine biosynthesis pathway and c-di-AMP favoring persistence-related *in vitro* phenotypes and persistence outcomes in VAN therapy of MRSA endovascular infections *in vivo*. This coordinated network offers novel therapeutic targets and strategies needed to address the growing threat of persistent MRSA infections and suggests the existence of a previously unknown adaptive genetic mechanism contributing to persistent MRSA infections.

## MATERIALS AND METHODS

### Bacterial strains, plasmids, and growth medium.

MRSA USA300 strain LAC and its derivative JE2 (cured of three plasmids) (40) were used as parental strains. The JE2 *purF* mutant from the Nebraska Transposon Mutant Library (NTML) and the LAC *dacA_G206S_* mutant in which the glycine at amino acid position 206 is replaced with a serine (6, 8) were also used. It has been reported that the *dacA* deletion mutant has a severe growth defect; thus, the *dacA* point mutant strain that grows robustly in rich medium (e.g., Trypticase soy broth [TSB]), while producing decreased c-di-AMP, was chosen for use in this study (6, 8). The *purF* and *dacA_G206S_* mutants were complemented by transforming plasmid pSK236::*purF* and pCL55::*dacA*, respectively, as described previously (6, 13). Unless otherwise stated, all S. aureus study strains were grown at 37°C in TSB (Difco) or on TSB agar plates.

### Determination of VAN MICs.

MICs of VAN on the study MRSA strains were determined by standard Etest method according to the manufacturer’s recommended protocols (bioMérieux, La Balme-les-Grottes, France).

### ATP and ADP levels.

ATP and ADP levels of study strains from overnight cultures were quantified by using Promega BacTiter Glo kit and Promega ADP-Glo kinase kit (Promega, Madison, WI), according to the manufacturer’s instructions, respectively (41, 42). ATP and ADP levels were determined by measuring luminescence levels compared to ATP and ADP standard curves, respectively, and presented as the levels normalized to CFU.

### Purification of c-di-AMP binding protein, CabP.

Escherichia coli strain ST2789, containing pET28a(+) with the *cabP* open reading frame (ORF) in E. coli strain BL21, was used to purify CabP protein according to the method described previously (43, 44). Briefly, the expression of CabP was induced by adding isopropyl-β-d-1-thiogalactopyranoside (IPTG) at 1 mM to the LB culture (30°C) at an optical density at 600 nm (OD_600_) of 0.5. Three hours after induction, E. coli cells were harvested and resuspended in lysis buffer (50 mM Tris-HCl [pH 7.5], 500 mM NaCl, 10 mM imidazole, and 10% glycerol). After sonication, bacteria debris was removed by centrifugation (20,000 × *g*) at 4°C. The CabP protein in the supernatant was purified by using a Capturem His-tagged purification miniprep kit (TaKaRa Bio USA, Ann Arbor, MI). The purity of the purified CabP protein was determined by SDS-PAGE. The concentration of the purified CabP protein was determined with a Pierce bicinchoninic acid (BCA) protein assay kit (Thermo Scientific).

### Detection of c-di-AMP levels.

c-di-AMP levels in the study MRSA strains were detected using a competitive enzyme-linked immunosorbent assay (ELISA) method as published previously (44, 45). Prior to determining the MRSA intracellular c-di-AMP levels, a standard curve was generated to calibrate the ELISA by using serial samples containing 25 nM biotin-labeled c-di-AMP and 2-fold serially diluted artificial c-di-AMP (ranged from 250 nM down to 7.8 nM) in Tris-HCl (45). MRSA cells from 10-ml overnight cultures were adjusted at an OD_600_ of 1.0, harvested, and resuspended in 500 μl of 50 mM Tris-HCl (pH 8.0). Following sonication and boiling, bacterial debris was removed by centrifugation for 5 min at 20,000 × *g*, and the supernatant was used to detect c-di-AMP levels. A 96-well plate was coated with CabP protein at 10 μg/ml at 4°C for at least 14 h. After washing and blocking the plates with 1% bovine serum albumin (BSA) for 1 h, biotin-labeled c-di-AMP (25 nM; Biolog) was added and incubated for 2 h. Then, the plate was washed and incubated with horseradish peroxidase-conjugated streptavidin (Thermo Scientific) for 1 h. The peroxidase was detected with the substrate *o*-phenylenediamine dihydrochloride (OPD) (Sigma) and measured at OD_492_.

### Biofilm formation.

Biofilm formation under static conditions with/without the addition of artificial c-di-AMP (InvivoGen, San Diego, CA) exposure was performed as previously described (28, 46). The adhering dye (0.1% safranin) was dissolved in 30% acetic acid, and absorption was measured as OD_490_ to quantify biofilm formation (28, 46).

### Detection of eDNA levels.

eDNA levels of overnight cultured study MRSA cells were detected by using a SYTOX green nucleic acid stain (Thermo Scientific) (19, 47). Briefly, 100 μl of filtered supernatant from the overnight cultures was mixed with 100 μl of 2 μM SYTOX green nucleic acid stain. Fluorescence was measured by using a BioTek Synergy 2 plate reader (BioTek, Winooski, VT, USA) with excitation and emission wavelengths of 465 nm and 510 nm, respectively. eDNA levels were expressed as relative fluorescence units (RFU).

### Lytic assays with Triton X-100 and VAN.

Lytic assays were performed as described elsewhere (48, 49). In brief, S. aureus cells from overnight cultures with/without c-di-AMP exposure were adjusted to an OD_580_ of 1.0, washed, and then exposed to 50 mM Tris-Cl (pH 7.2) containing 0.1% Triton X-100 or 20 μg/ml (10× MIC of VAN) and incubated at 30°C with agitation (200 rpm). Staphylococcal lysis was measured by the changes in OD_580_.

### VAN binding to MRSA.

VAN binding to study MRSA strains was measured using a boron dipyrromethene difluoride-labeled VAN strategy (Bodipy FL VAN; Invitrogen Corp., Carlsbad, CA) (46). Briefly, overnight cultured S. aureus cells were adjusted to an OD_600_ of 1.0 and then exposed to Bodipy FL VAN at the concentration of 20 μg/ml (10× MIC of VAN) for 30 min at 37°C in cation-adjusted Mueller-Hinton broth (MHB). The binding of VAN was measured by quantitative flow cytometry (FACSCalibur; Becton, Dickinson [BD]) (46, 50). For each sample, 10,000 cells were acquired and analyzed. The results were expressed as the percentage of acquired cells exhibiting threshold levels of the fluorescence signal.

### Detection of WTA in MRSA.

Staphylococcal cell wall GlcNAc, one of the important WTA structural components, was quantified by using a wheat germ agglutinin (WGA)-Alexa Fluor 594 conjugate (Invitrogen) (51, 52). Alexa Fluor 594 WGA selectively binds to GlcNAc substituents in WTA on the surface of S. aureus (53). Briefly, overnight cultured S. aureus cells with/without c-di-AMP exposure were adjusted to an OD_600_ of 1.0, washed, and resuspended within 1 ml phosphate-buffered saline with Tween 20 (PBST) buffer (120 mM NaCl, 50 mM phosphate, 0.1% Tween 20, pH 8.0). The 100-μl samples were mixed with 50 μl of Alexa Fluor 594 WGA solution (100 μg/ml) and incubated for 10 min at room temperature. After washing with a PBST buffer, fluorescence was measured by using a BioTek Synergy 2 plate reader (BioTek, Winooski, VT, USA) with excitation and emission wavelengths of 590 nm and 617 nm, respectively. GlcNAc levels were expressed as RFU.

### RNA isolation and real-time quantitative reverse transcription-PCR.

Total RNA for *dacA*, *gdpP*, *lytS*, *lytR*, *lrgB*, and *tarH* expression was isolated from overnight cultured study MRSA strains (same incubation time as the experiments above for measuring ADP, ATP, and c-di-AMP levels) by using an RNeasy kit (Qiagen, Valencia, CA) (18). Then DNase-treated RNA was transcribed into complementary DNA. Real-time quantitative reverse transcription-PCR (RT-qPCR) was performed using an ABI Prism 7000 instrument (Applied Biosystems) and a SYBR green PCR master kit (Applied Biosystems). The primers for *dacA*, *gdpP*, *lytS*, and *lytR* amplification have been described previously (11, 54). The primers used to amplify *lrgB* and *tarH* were *lrgB*-F (5′-ACAATCTGTTTTGCGATTCCG-3′) and *lrgB*-R (5′-CTGTAGTTGCTGCTTGAGGT-3′) (23) and *tarH*-F (5′-TAACGAAGCGGGACTCATCG-3′) and *tarH*-R (5′-TGCTTGGATTGAAGGCGGAA-3′), respectively. *gyrB* was used to normalize the transcript quantification, and relative expression of the study genes was calculated by the ΔΔ*C_T_* method (3).

### Experimental endocarditis model in rabbits.

A well-characterized rabbit model of catheter-induced aortic valve endocarditis was used to study the composite metrics of virulence and responsiveness to VAN therapy among the MRSA LAC parental strain and its isogenic *dacA_G206S_* mutant and *dacA*-complemented strains (18, 28). The Institutional Animal Care and Use Committee of the Lundquist Institute at Harbor-UCLA Medical Center approved all animal study protocols. After 72 h of catheterization, animals were infected intravenously (i.v.) with the LAC parental strain, its *dacA_G206S_* mutant, or *dacA*-complemented strain at 10^5^ CFU/animal, an 95% infective dose (ID_95_) as established previously (18, 28). At 24 h postinfection, animals were randomized to receive no therapy (controls) or VAN (3.75 mg/kg of body weight, i.v., twice daily for 3 days; this dose-regimen of VAN was shown to exert limited microbiologic clearance of the parental strain from any target tissue based on extensive pilot studies). Control animals were sacrificed at 24 h postinfection in order to determine MRSA density in target tissues at the beginning of VAN treatment. VAN-treated animals were sacrificed 24 h after the last treatment dose to avoid VAN carryover effect. At sacrifice, cardiac vegetation, kidney, and spleen were sterilely removed and quantitatively cultured (18, 28). MRSA counts in the target tissues were given as the mean log_10_ CFU per gram of tissue (± standard deviation [SD]).

### Statistical analysis.

All *in vitro* experiments were performed in triplicate and repeated at least twice. Statistical significance values of *in vitro* and *in vivo* experiments were obtained by performing a two-tailed Student's *t* test and one-way analysis of variance (ANOVA) with Tukey’s multiple-comparison test (no adjustment), respectively. *P* values of <0.05 were considered statistically significant.
